# Supplementary material for: metaGWASmanager: a toolbox for an automated workflow from phenotypes to meta-analysis in GWAS consortia
Source: Bioinformatics. 2024 Apr 30;40(5):btae294. doi: 10.1093/bioinformatics/btae294 (PMC11096268; doi:10.1093/bioinformatics/btae294)
Supplement: btae294_Supplementary_Data [file btae294_supplementary_data.pdf]

Overview

This vignette introduces the metaGWASmanager, a comprehensive toolbox leveraging existing software packages and, streamlining the entire GWAS-consortium workflow. This encompasses the phenotype generation, quality control of phenotypes, GWAS, GWAS-QC and meta-analysis providing an integrated solution for both the participating Study Analysts (SA) and Consortium Analysts (CA). For a description, please see the corresponding manuscript: Zulema Rodriguez-Hernandez, Mathias Gorski, Maria Tellez Plaza, Pascal Schlosser* and Matthias Wuttke* (2023). “metaGWASmanager: A toolbox for an automated workflow from phenotypes to meta-analysis in GWAS consortia”. Under review.

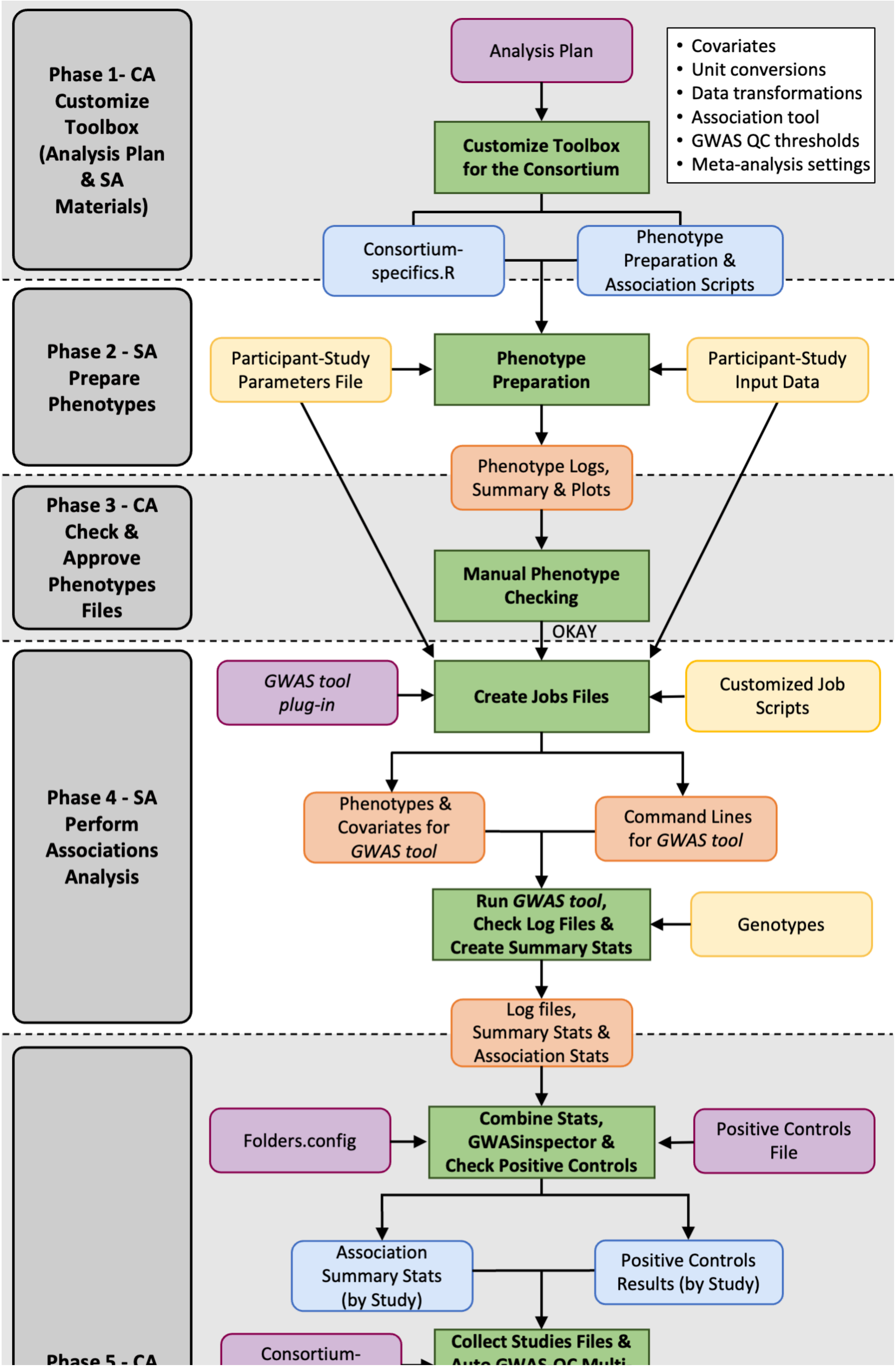
Phase 1- CA Customize Toolbox (Analysis Plan & SA Materials)
Analysis Plan
Customize Toolbox for the Consortium
Covariates
Unit conversions
Data transformations
Association tool
GWAS QC thresholds
Meta-analysis settings
Consortium-specifics.R
Phenotype Preparation & Association Scripts
Phase 2 - SA Prepare Phenotypes
Participant-Study Parameters File
Phenotype Preparation
Participant-Study Input Data
Phenotype Logs, Summary & Plots
Phase 3 - CA Check & Approve Phenotypes Files
Manual Phenotype Checking
OKAY
Phase 4 - SA Perform Associations Analysis
GWAS tool plug-in
Create Jobs Files
Customized Job Scripts
Phenotypes & Covariates for GWAS tool
Command Lines for GWAS tool
Run GWAS tool, Check Log Files & Create Summary Stats
Genotypes
Log files, Summary Stats & Association Stats
Folders.config
Combine Stats, GWASinspector & Check Positive Controls
Positive Controls File
Association Summary Stats (by Study)
Positive Controls Results (by Study)
Consortium-
Collect Studies Files &

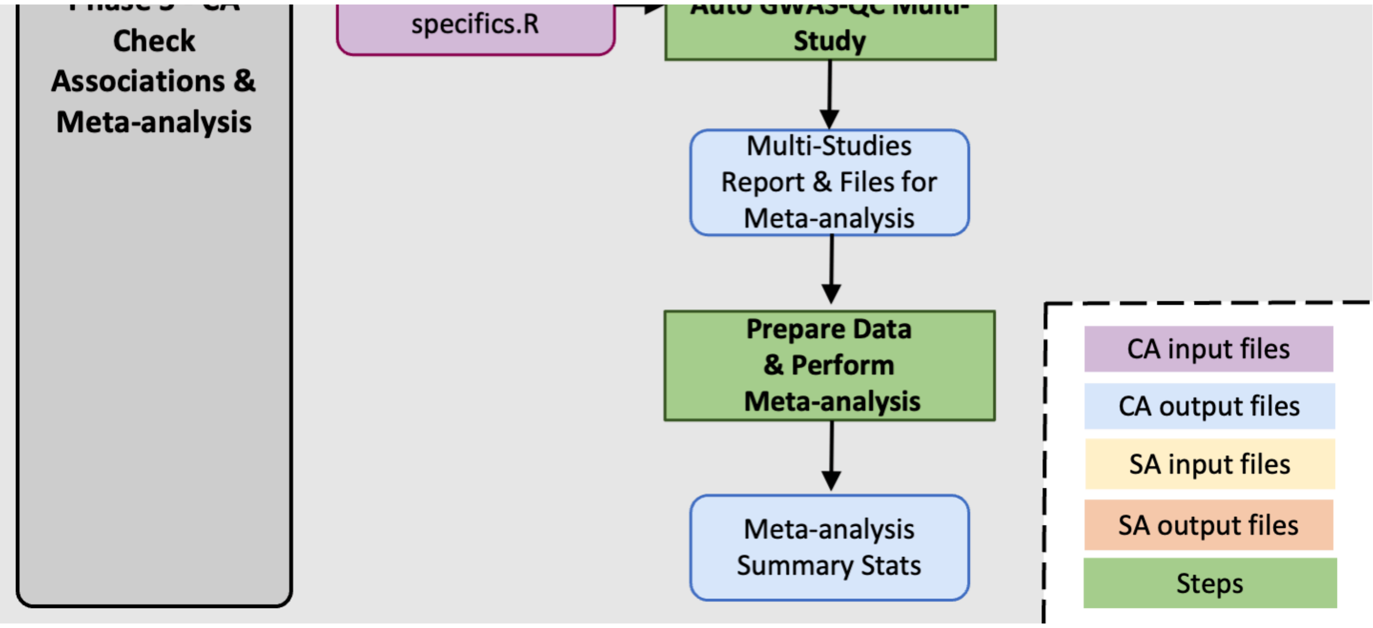

Illustration of the metaGWASmanager pipeline, outlining phenotype generation and quality assurance, followed by the GWAS, GWAS-QC and meta-analysis steps, along with the required inputs and resulting outputs. Shaded and white sections indicate tasks to be carried out by CA and SA, respectively.

Installation

To set up the toolbox please fork the github repository: https://github.com/genepi-freiburg/gwas-consortium/tree/main

Required files, programs and server specifications

Aside from the documentation and scripts supplied by metaGWASmanager, it is necessary to ensure that the following programs and files are properly installed and downloaded on your system.

For SAs

- Association tool to use. (e.g., regenie)
- PLINK or PLINK 2.0
- VCFtools and BCFtools
- R software
- Analysis plan, scripts and examples files for conducting Phases 2 and 4 will be provided by the CAs.

For CAs

Same as SAs, but also:

- GWASinspector R package
  - SQLite reference data available here
  - The alt_headers.txt file is available in the extended data in the CRAN Archive
- Perl
- HTSlib

- FlexiBLAS
- Python

Server structure

We outline the recommended structure for the consortium server to ensure effective and efficient data management:

Scripts

They should be separate from data, in a different folder.

```
"/storage/consortium_name/scripts/"
```

This folder will contain the scripts needed by CAs, all provided by metaGWASmanager. It is advisable to directly download the different folders-containing scripts (*pheno_generation*, *pheno_summary*, *gwas_qc* and *metaanalysis*) from metaGWASmanager GitHub to the *scripts* folder previously created on your server:

- **pheno_generation** folder: it contains scripts needed by SAs to prepare phenotype data, and perform GWAS. To be used by SAs.
- **pheno_summary** folder: it contains scripts to summarize phenotype submissions across studies. For CAs' use.
- **gwas_qc** folder: it contains scripts to summarize genotype submissions across studies. For CAs' use.
- **metaanalysis** folder: it contains scripts to prepare data and perform meta-analysis. For CAs' use.

Please, do not modify the scripts names.

Pheno Upload

The phenotypes results, output of **Phase 2 - SA: Prepare Phenotypes (SAs)**, submitted by the SAs will be stored in the *uploads/pheno* directory. This directory will contain a folder for each study that has submitted the phenotype summary statistics.

```
"/storage/consortium_name/uploads/pheno/study_name"
```

Besides the study-specific folders, it should also contain two additional directories:

- *00_ARCHIVE*. If a specific-study re-upload the phenotype summary statistics, perhaps due to the detection of an error, CAs will archive the previous version in this directory.
- *00_SUMMARY*. The output from performing cross-study phenotype summaries, in the **Phase 3 - CA: Check & Approve Phenotypes Files**, will be stored in this location.

GWAS Upload

The GWAS results (output of **Phase 4 - SA: Perform Associations Analysis**) submitted by the SAs will be stored in the *uploads/assoc* directory. This directory will contain a folder for each study that has submitted genetic summary statistics.

```
"/storage/consortium_name/uploads/assoc/study_name"
```

Similar to the *uploads/pheno* directory, it is recommended to create a *00_ARCHIVE* sub-directory.

It is advised to maintain consistency in the naming of studies within both the *uploads/pheno* and *uploads/assoc* folders to prevent potential problems when cross-referencing files between the two folders.

Cleaning

Contains intermediate files, logs and summary files resulting from **Phase 5 - CA: Check Associations & Meta-analysis**.

```
"/storage/consortium_name/cleaning/study_name"
```

In addition to the folders designated for each study, it should also encompass two extra directories:

- *00_ARCHIVE*. To save older results.
- *00_SUMMARY*. It will contain the output of cross-study GWAS verification process (**Phase 5 - CA: Check Associations & Meta-analysis**) including plots, overall statistical summaries, positive controls validation result, among others.

Note that the sub-folder "*data*" within each study folder contains the GWAS results combined by chromosomes.

```
"/storage/consortium_name/cleaning/study_name/data"
```

Meta-analysis

Finally, the meta-analysis folder will contain as many subfolders as target traits (e.g, uacr_int trait).

```
"/storage/consortium_name/metaanalysis/uacr_int"
```

Step by step guide

Phase 1 - CA: Customize Toolbox (Analysis Plan & SA Materials)

The initial stage involves generating Consortium-specific files (*consortium-specifics.R* and *parameters.txt* plug-in) and formulating the Analysis Plan, in which CAs will define, based on the provided metaGWASmanager examples, the required settings for conducting the pool-cohort GWAS.

A) ***consortium-specifics.R***. The *consortium-specifics.R* plug-in file (located in the *pheno_generation* folder) should be edited according to the CA specifications. This file contains several functions that allow customization of the entire analysis process: verification of the *input* and *parameteres* files; unit conversion; traits transformations (rank-based inverse normalization and log-transformation are implemented); setting quality control parameters, covariates and stratification-variables; association tool to be used (e.g., *PLINK 2.0*, *regenie*, etc), among others. These functions will be applied in subsequent stages of the workflow process. The ***consortium-specifics.R*** file will be provided to each SAs along the Analysis Plan and required scripts.

Please, note that, regarding the type of association tool to use, currently metaGWASmanager has implemented *PLINK 2.0* and *regenie* softwares. CAs can choose one of these two. However, in case CAs prefer to use another different one, we provide a plug-in interface, ***assoc_tool_plugin.R***, within the *pheno_generation/assoc_tool* folder, that CAs can customize to add a different association tool.

B) **Parameters file**. CA will customize the ***parameters.txt*** file based on the particular traits to study. It must included important points, which must be filled out by the SAs, such as the name of the input file and the

participant-study, SAs contact information, the study's ancestral background, analysis date, units of the variables to be studied, laboratory particular settings (e.g., limits of detection), imputation reference panel, number of genetic principal components, and some more optional fields. A parameters file example is provided within the *pheno_generation* folder on the metaGWASmanager GitHub repository.

C) **Analysis Plan**. The Analysis Plan (e.g., PDF document) should provided comprehensive information for SAs on how to proceed with the different analyses, including **Phase 2 - SA: Prepare Phenotypes** and **Phase 4 - SA: Perform Associations Analysis**. Certainly, you are welcome to use the information described in this documentation. It is also essential to include CAs contact details in case any questions arise.

Phase 2 - SA: Prepare Phenotypes

In a first step, CA will ask for preparation the phenotypic data for all participating studies. CA will supply an Analysis Plan and the *pheno_generation* folder. Please note, that for each study and for each ancestry analyzed, one copy of the *pheno_generation* folder should be used.

Input files for each participating study include: a) the ***input.txt*** file, a database created by SA according to the CA guide standards (see the ***dummy-input.txt*** example), and b) SAs will complete the parameter file (***parameters.txt***) provided by CAs.

After that, SAs will execute the ***01-phenotypes-generation.sh*** script, which automatically generates the descriptive statistics of phenotypes, on a Linux server as follows:

```
bash 01-phenotypes-generation.sh parameters.txt
```

Note that if SAs prepare their data in a Windows or Mac system, to avoid problems with line breaks when running the script in Linux/Unix, they should use the following commands, respectively, to convert the files prior to running the script:

```
dos2unix input.txt
```

```
mac2unix input.txt
```

During this initial phase, a comprehensive examination of the input and parameters files will be carried out. This involves issuing warnings and identifying errors to guarantee high-quality phenotypic data and prevent common pitfalls such as unit conversion discrepancies or variations in assay methods. Additionally, the pipeline executes essential trait transformations (e.g., rank-based inverse normalization, log-transformation) and calculations, ensuring uniformity in the phenotype data.

The files generated by the phenotype preparation scripts include the phenotype summary statistics (***STUDY_NAME_ids_summary.txt***) and plots files. SAs must carefully examine the *.log* files for errors or warnings, as well as ensure that the generated descriptive statistics and plots (PDF file) are reasonable. If there are problems, SAs will try to adjust the parameters in the ***parameters.txt*** or ***input.txt*** file according to the different error messages.

In case everything seems fine, SAs will send all the files from the generated *return_pheno* folder via email to CAs. The CAs will then check phenotype descriptive data. It is important to note that the files uploaded by SAs contain only summarized statistical (not individual-level information).

You can find an example of a phenotype generation report here. It includes descriptive graphs (such as box plots and histograms) for the trait-specific variables (genetic principal components, outcomes, covariates, and stratified variables). When applicable, an informative bar plot is provided for each binary and categorical variable.

Phase 3 - CA: Check & Approve Phenotypes Files

The submited files within the *return_pheno* folder will be stored in the *upload/pheno* directory by CAs, within the folder corresponding to the specific participating study.

```
"/storage/consortium_name/uploads/pheno/study_name"
```

Subsequently, CA will manually inspect these files and plots in order to identify potential issues and inconsistencies, such as anormal distributions ando/or outliers. Furthermore, CA will run the scripts located in the *pheno_summary* directory of the metaGWASmanager toolkit (***01_collect_summary.sh*** and ***02_plot_summaries.sh***).

Both scripts summarize phenotype summary-statistics submissions (***STUDY_NAME_ids_summary.txt***) across studies and also facilitate their representation for improved inter-study comparison and to detect potential outliers. Please run both scripts using the *bash* command line from *pheno_summary* folder:

```
"/storage/consortium_name/scripts/pheno_summary"
```

Note that the generated outputs of the inter-study comparison analyses will be automatically saved in the *00_SUMMARY* folder located at:

```
"/storage/consortium_name/uploads/pheno/00_SUMMARY"
```

After the inspection of the submitted files, CA will provide feedback to SAs. In the case CA detects an issue, it will be clearly outlined and assistance will be offered to the SAs in oder to resolve it. The SAs will then revisit **Phase 2 - SA: Prepare Phenotypes** and re-submit the corresponding outputs files. Contrary, if no problems are identified, SAs are authorized to proceed with **Phase 4 - SA: Perform Associations Analysis**.

Phase 4 - SA: Perform Associations Analysis

To initiate this step, SAs should have received the approval from CA and they must have prepared the genetic information.

For both association tools (*PLINK 2.0* and *regenie*, specially for *regenie*-step2) imputed genetic data (chunked by chromosome) in *.bgen* format v1.2 with 8 bits encoding is required. SAs can easily convert the imputed data to *.bgen* format using the provided ***vcf_to_bgen.sh*** script, inside the *pheno_generation* folder or by running the following commands on Linux:

```
plink2 --vcf $infile dosage=DS --make-pgen erase-phase --out $pgen_unphased
```

```
plink2 --pfile $pgen_unphased --export bgen-1.2 bits=8 --out $out_bgen
```

For the *regenie* association tool, the measured genotype (chip data) in PLINK format is also required for the *regenie*-step1. We recommend to extract the high quality variants following the *regenie* best practices:

```
plink2 --geno 0.1 --hwe 1e-15 --mac 100 --maf 0.01 --mind 0.1 --indep-pairwise 1000
100 0.9
```

For that, SAs can use the provided ***plink_qc.sh*** script.

Once the genetic data is prepared, the SAs can systematically initiate the necessary script executions.

A) **Create job files**.

First, SAs should adjust the *make-association-job-scripts* within the *pheno_generation/assoc_tool* folder according to their requirements (data path and genotype file prefix, etc).

A.1) **For regenie:**.

Two files to be modified by SAs: ***make-regenie-step1-job-scripts.sh*** and ***make-regenie-step2-job-scripts.sh***:

For ***make-regenie-step1-job-scripts.sh***:

```
. REGENIE=<path to your regenie software>
. PLINK_DATA_PREFIX=<path and prefix to your PLINK *.bed file>
. PLINK_SNP_QC=<path to your *.snplist file containing a list of high quality variants used for
regenie-step1>
. PLINK_INDIV_QC=<path to your *.id file containing a list of high-quality individuals used for
regenie-step1>
. --threads <Number of threads that should be utilized by each individual regenie-step1 job>
```

For ***make-regenie-step2-job-scripts.sh***:

```
. REGENIE=<path to your regenie software>
. BGEN_SAMPLE_PREFIX=<path to your *.bgen and *.sample files> (as one sample file exists per
chromosome; please adjust the prefix file name using ${CHR} as placeholder for the chromosome).
. --threads <Number of threads that should be utilized by each individual regenie-step-2 job>
```

A.2) **For PLINK 2.0:**.

One file to be edited by SAs: **make-plink-job-scripts.sh**:

```
. PLINK=<path to your PLINK 2.0 software>
. BGEN_SAMPLE_PREFIX=<path to your *.bgen and *.sample files> (as one sample file exists per
chromosome; please adjust the prefix file name using ${CHR} as placeholder for the chromosome).
. --threads <Number of threads that should be utilized by each individual plink job>
```

Note that these scripts are set up to utilize a Slurm job scheduler.

Upon making the mentioned adjustments, SAs can executed the ***02-consortium-make-assoc-jobs.sh*** script.

```
bash 02-consortium-make-assoc-jobs.sh parameters.txt
```

The process will generate the different jobs (saved in the *jobs* folder), which include the phenotypes and covariates to be used along with the command lines necessary to run the association analyses.

For instance, for *regenie*-step1, as many jobs as phenotype will be created, while one job for each phenotype and chromosome for *regenie*-step2 and *PLINK 2.0*.

B) **Run GWAS**

SAs then will execute ***03-submit-all-jobs.sh*** to submit all GWAS according to the jobs generated in the preceding step.

```
bash 03-submit-all-jobs.sh parameters.txt
```

Our recommendation is to first run a pilot analysis before submitting all jobs. For example, if *regenie* is used, one phenotype for *regenie*-step1 and one chromosome for *regenie*-step2.

C) **Create Summary Statistics** and **Collect & Upload results**.

The SAs will then run the ***04-postprocess-results.sh*** script, which investigate the different log files generated during the association process to ensure the successful execution of each GWAS. It also produces tailored summary tables for each association analysis.

```
bash 04-postprocess-results.sh parameters.txt
```

If an issue occurs, SAs should investigate the cause. After resolving it, SAs can re-execute individual phenotypes and/or steps by using the appropriate files from the *jobs* directory. In case SAs needed to change any parameter o paths, they would have to re-run the ***02-consortium-make-assoc-jobs.sh*** again.

Finally, the results will be compiled into a compressed folder using ***05-collect-files-for-upload.sh***

```
bash 05-collect-files-for-upload.sh study_name
```

It will be created a a gzipped tar-ball folder containing outputs from *return_pheno* folder including log files, summary tables and plots, and the association-output folders (*regenie-step2* or *output_plink* folder). The compressed folder will then be submitted to the CA server for further validation.

We also recommend that CAs, at this point, somehow collect information about the specific authors (names, affiliations, conflict of interest, etc) and studies (genotyping information, study acknowledgements, other relevant study information). For example, CAs may use a Google Sheets and/or Google Forms.

Phase 5 - CA: Check Associations & Meta-analysis

The compressed folder submitted by the SAs, will be stored in the *upload/assoc* directory, within the folder corresponding to the specific participating study.

```
"/storage/consortium_name/uploads/assoc/study_name"
```

CA will then proceed to unzip the folder and run scripts located in the *gwas_qc* folder of the metaGWASmanager toolbox. Please, note that scripts located in *gwas_qc* will be run directly from this folder.

```
"/storage/consortium_name/scripts/gwas_qc"
```

folders.config.sh **file**

It includes details about essential pathways (script folder, cleaning directory, upload data, etc) and specific variables (phenotypes, ancestry and strata vectors, among others) needed for executing downstream scripts. CAs first should customize these fields and may also incorporate important settings to align with particular server requirements. The ***folders.config.sh*** file will be sourced throughout subsequent scripts, so no modification to other scripts are needed.

After making the necessary settings to the *folders.config.sh* file, CA are ready to carry out the subsequent steps.

A) ***01_combine_chromosomes.sh***.

It checks if any files are missing from the association-output folder ensuring the presence of the required columns using the **find-column-index.pl** script. Subsequently, it merges all the chromosomes into a single compressed file for each trait and the corresponding tabix files are generated.

Command line to run:

```
bash 01_combine_chromosomes.sh study_name
```

B) ***02_gwasinspector.sh***.

CAs will then run GWASinspector:

```
bash 02_gwasinspector.sh study_name
```

GWASinspector performs an extensive quality control process on GWAS results ensuring their accuracy, proper format, and consistency across all studies using robust criteria. As a result, in the *cleaning/study_name/qc_output* folder, clean and harmonized files will be created along with a comprehensive report and plots (including Manhattan, QQ plots, histograms, etc) as shown below:

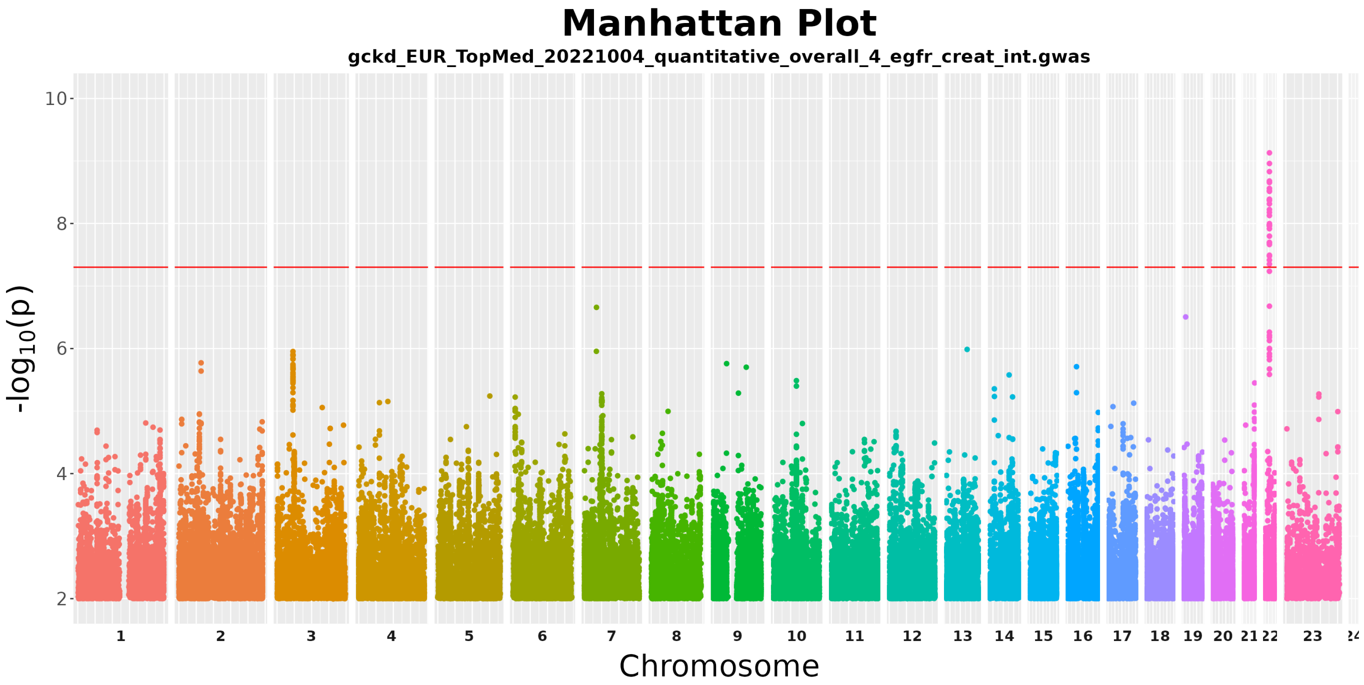

Manhattan plot created by GWASinpector workflow of overall eGFR creatinine in the GCKD cohort, a participating study in the CKDGenR5 consortium.

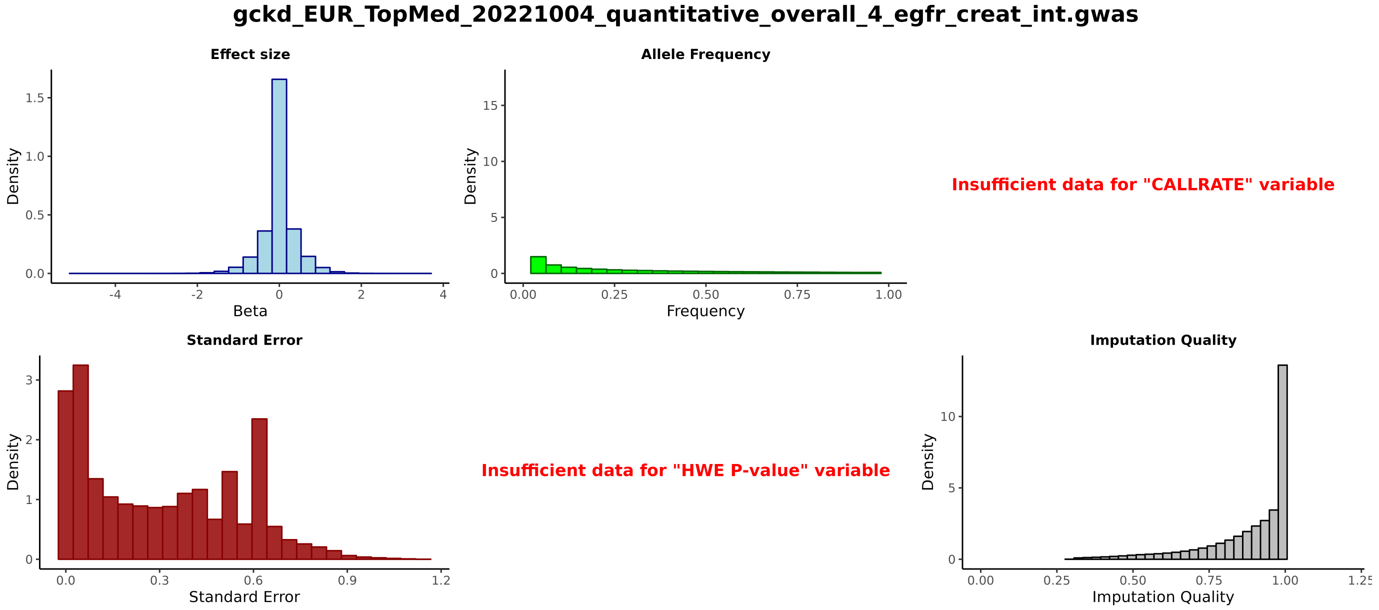

Quality controls plot created by GWASinpector workflow of overall eGFR creatinine in the GCKD cohort, a participating study in the CKDGenR5 consortium.

More information about GWASinspector R package here.

C) ***03_check_positive_controls.sh***

CAs should create a GWAS positive control *.txt* file containing the hits (specific to each target trait and ancestry) widely validated in the literature, whenever possible.

CAs can also find an example in the *gwas_qc/Positive_Controls* folder (please, replace the provided example by your own customized ***positive-controls.txt*** file):

trait	ethnicity	SNP	gene	chr	pos_b37	pos_b38	ref	alt	MA[illegible]
egfr_creat_int	EUR	rs13329952	UMOD	16	20366507	20355185	T	C	0.1[illegible]
egfr_creat_male	EUR	rs13329952	UMOD	16	20366507	20355185	T	C	0.1[illegible]
egfr_creat_female	EUR	rs13329952	UMOD	16	20366507	20355185	T	C	0.1[illegible]
egfr_creat_int	EAS	rs10277115	UNCX	7	1285195	1245559	A	T	0.3[illegible]
egfr_creat_male	EAS	rs10277115	UNCX	7	1285195	1245559	A	T	0.3[illegible]
egfr_creat_female	EAS	rs10277115	UNCX	7	1285195	1245559	A	T	0.3[illegible]

By running ***03_check_positive_controls.sh***, each file within the *cleaning* directory will be checked and compared with the created ***positive-controls.txt***

```
bash 03_check_positive_controls.sh study_name
```

Results will be saved in ***03-check-positive-controls.csv*** file within the corresponding folder:

```
"/storage/consortium_name/cleaning/study_name"
```

D) ***04_collect_qc_stats.sh***

It checks all clean studies after GWASinspector quality control:

```
"/storage/consortium_name/cleaning/study_name/qc_output"
```

and generate a ***qc-stats.csv*** and ***qc-stats.xlsx*** files in the *00_SUMMARY* folder:

```
"/storage/consortium_name/cleaning/00_SUMMARY"
```

The ***qc-stats.csv*** contains information about the specific study, phenotype, ancestry, sample size, variants per chromosome, lambda and summary stats for both "All" and "High quality" variants, such as P-value (PVAL), effect allele frequency (EFF_ALL_FREQ), imputation quality (IMP_QUALITY), effect size (BETA), standard error (STDERR). The file then, will contain as many rows as specific studies and phenotypes.

STUDY	PHENO	POP	PVALUE_MED_ALL	EFF_ALL_FREQ_MED_ALL	IMP_QUALITY_MED_
GCKD_2022-10-04	ckd	EUR	0.4822	0.001215	0.9469
GCKD_2022-10-04	ma	EUR	0.4787	0.001851	0.9572
GCKD_2022-10-04	gout	EUR	0.4743	0.001198	0.9466

Command line to run:

```
bash 04_collect_qc_stats.sh
```

In addition, this script generates a general (all specific studies) positive controls summary files (***positive-controls.csv*** and ***positive-controls.xlsx***) in the *00_SUMMARY* folder.

E) ***05_plot_qc_stats.sh***

Multi-studies summary plots will be then generated from ***qc-stats.csv*** file within the *00_SUMMARY/plots* folder:

```
"/storage/consortium_name/cleaning/00_SUMMARY/plots"
```

Command line to run:

```
bash 05_plot_qc_stats.sh
```

F) ***06_plot_frequencies.sh***

Running ***06_plot_frequencies.sh*** generates a correlation plot for each phenotype across all the specific studies. The objective is to verify the correlation of allele frequencies between the observed data with a reference panel.

```
bash 06_plot_frequencies.sh
```

Plots will be save in study-specific folder.

```
"/storage/consortium_name/cleaning/study_name/freqs"
```

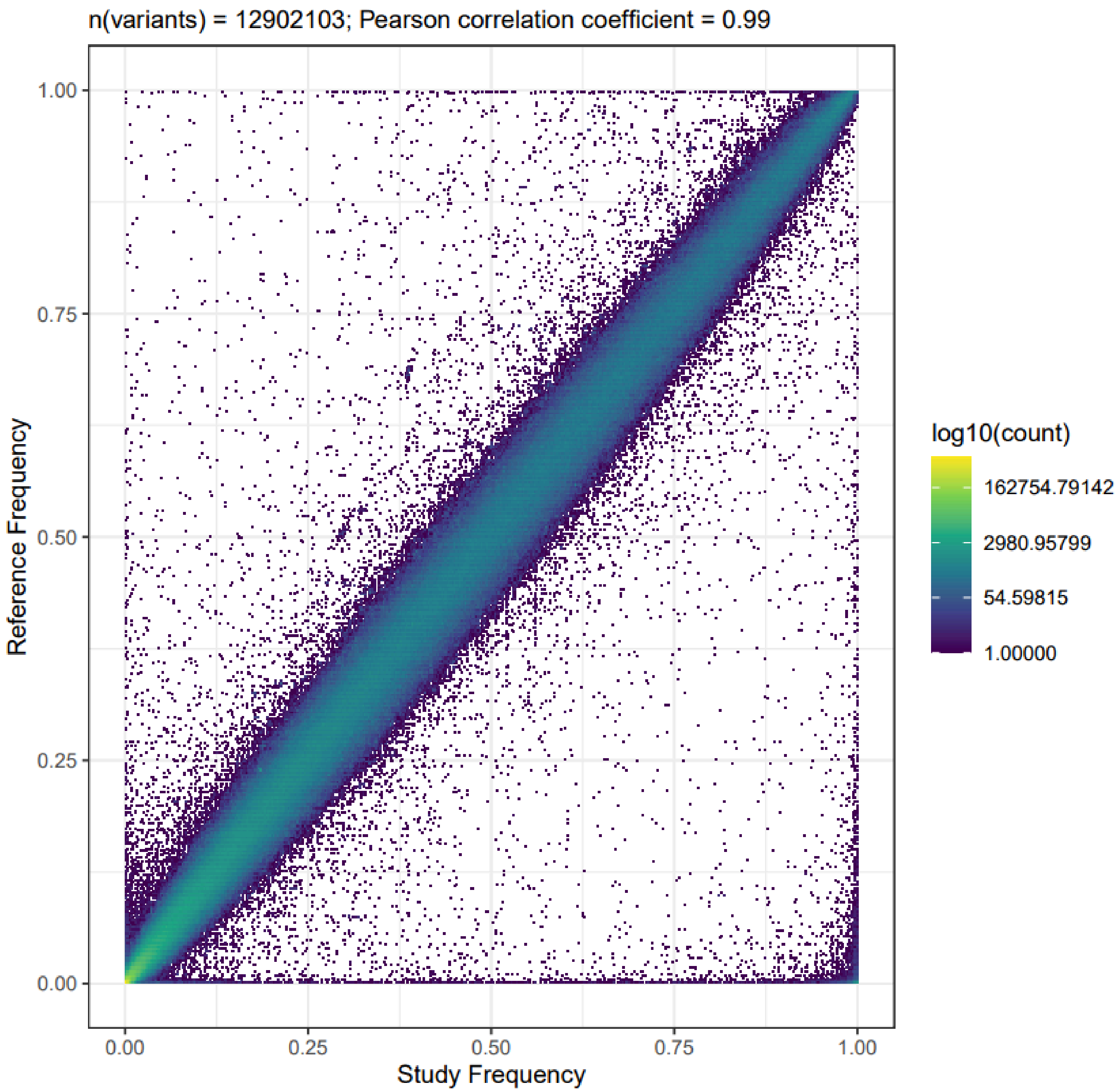

Frequency correlation plot of overall eGFR creatinine in the GCKD cohort, a study participating in the CKDGenR5 consortium.

G) ***07_stateOfAffairs.sh***

Efficient monitoring of the consortium's progress, such as ongoing GWAS status, studies awaiting analysis, and current sample sizes is crucial for effective coordination and management of the consortium effort. The ***07_stateOfAffairs.sh*** print such report and a pie plot from ***qc-stats.csv*** file.

Command line to run:

```
bash 07_stateOfAffairs.sh
```

H) ***08_checkAllFileNames.sh***

For proper execution of subsequent steps (Auto-GWAS quality control and meta-analysis) a final check is important to ensure consistency in files names, studies and phenotypes. The ***08_checkAllFileNames.sh*** scripts systematically validate these aspects and report if any inconsistencies arise.

```
bash 08_checkAllFileNames.sh
```

I) ***09_GWAS_QC_multistudies.sh***

By combining the summary statistics from GWAS QC (***qc-stats.csv***) and the results of positive controls analysis (***positive-controls.csv***) across all participating studies, CA will perform a final checking process. A set of relevant items will be checked, including: wrong genomic build, allele switches and swaps, missing data, file and number formatting issues, unaccounted inflation, and wrong transformations, among others.

metaGWASmanager provides a script (***09_GWAS_QC_multistudies.sh***), which automatically evaluates the list of potential problems mentioned above. For its execution, CA will require the previously edited ***consortium-specifics.R*** plug-in, located in the *pheno_generation* folder. It supplies a tolerance table establishing the thresholds to apply at each verification stage based on the different phenotypes and covariates to be tested.

Command line to run:

```
bash 09_GWAS_QC_multistudies.sh
```

In the end, a conclusive-summary table will be generated in the *cleaning/00_SUMMARY/AutoQC* folder, indicating whether each trait for each assessed item is categorized as "OKAY" or "NOT OKAY".

study	pheno	pop	N	pval	lambda	impqual	beta	stderr	allel_
GCKD_2022-10-04	ckd	EUR	4993	OK	OK	OK	OK	OK	OK
GCKD_2022-10-04	ma	EUR	3998	OK	NOT OK	OK	OK	OK	OK
GCKD_2022-10-04	gout	EUR	5034	OK	NOT OK	OK	OK	OK	OK
GCKD_2022-10-04	albumin_serum_int	EUR	4992	OK	OK	OK	NOT OK	OK	OK
GCKD_2022-10-04	egfr_creat_int	EUR	4993	OK	OK	OK	OK	OK	OK

CA should scan all entries and emphasizing those labeled as "NOT OKAY", as well as, plots produced by GWASinspector step. If an explanation for any identified issues cannot be determined, CAs must reach out to the SAs to collaboratively work towards a resolution.

After completing all GWAS quality controls checks, CAs are pepared to proceed with the meta-analysis pipeline. The necessary scripts are located in the *metaanalysis* folder provided by metaGWASmanager, and they should be run here:

```
"/storage/consortium_name/scripts/metaanalysis"
```

J) ***01-locate-input-files.sh***

First, CAs must prepare the ***input-file-list.txt*** containing all the study names to be meta-analyzed as follows:

GCKD_2022-10-04
Study_name_2
Study_name_3
...
Study_name_N

The file should be save within the created *metaanalysis* directory.

```
"/storage/consortium_name/metaanalysis"
```

Given a phenotype (e.g., uacr_int) the ***01-locate-input-files.sh*** script creates the ***input-files-with-path.txt*** file with the full paths to the cleaned association-output files for the specific phenotype across all studies intended for meta-analysis.

```
bash 01-locate-input-files.sh uacr_int
```

K) ***02-prepare-ma-input.HQ.sh*** and ***02-prepare-ma-input.LQ.sh***

They are both mostly the same scripts, but the first with stringent filter settings and second with lenient filter settings. Based on the filter configurations, input files per specific phenotype and study will be generated in the *input* folder.

```
"/storage/consortium_name/metaanalysis/uacr_int/HQ/input"
```

Command line to run:

```
bash 02-prepare-ma-input.HQ.sh uacr_int
```

L) ***03-prepare-metal-params.sh***

The ***03-prepare-metal-params.sh*** script uses the ***metal-params.txt*** template, which contains different filters (MAF, MAC, genomic control [yes, or not], etc) to be set by CAs according to their preferences. The selected options are then used by the METAL software for conducting pior meta-analysis steps (e.g., genomic control correction) and meta-analysis.

To run the script, the phenotype (e.g., uacr_int) and type of selected variants (e.g, LQ or HQ) must be provided as arguments:

```
bash 03-prepare-metal-params.sh uacr_int HQ
```

By running this script, the configured ***metal-params.txt***, needed to perform meta-analysis, will be generated at:

```
"/storage/consortium_name/metaanalysis/uacr_int/HQ/metal_output_HQ"
```

M) ***04-run-metal.sh***

Finally, run meta-analysis as follow:

```
bash 04-run-metal.sh uacr_int HQ
```

Results will be stored in a *output* folder:

```
"/storage/consortium_name/metaanalysis/uacr_int/HQ/metal_output_HQ/output"
```

Here CAs will find a **.info** file, encompassing details about the meta-analysis process, parameters and input files used. Additionally, a *.tbl* file containing the meta-analysis results will be generated.

MarkerName	Allele1	Allele2	Freq1	FreqSE	MinFreq	MaxFreq	Effect	St
3:53066069:C:G	c	g	0.9993	0.0000	0.9993	0.9993	-2.59795000	3.27
3:100796605:A:G	a	g	0.9936	0.0003	0.9929	0.9937	0.10832363	0.101
1:174816432:C:T	t	c	0.0051	0.0015	0.0041	0.0073	-0.08297466	0.12
7:108043353:A:C	a	c	0.0002	0.0000	0.0002	0.0002	0.37409000	0.63
2:234097095:A:G	a	g	0.1593	0.0128	0.1519	0.1814	-0.00944623	0.02
5:128938723:G:T	t	g	0.2990	0.0011	0.2968	0.2995	-0.00445147	0.01
8:2498343:C:T	t	c	0.0002	0.0000	0.0002	0.0002	0.17083500	0.64

More information about the meta-analysis process here.
